# Supplementary material for: The Allelopathic Activity of Extracts and Isolated from Spirulina platensis
Source: Molecules. 2022 Jun 15;27(12):3852. doi: 10.3390/molecules27123852 (PMC9227131; doi:10.3390/molecules27123852)
Supplement: Supplementary file 1 [file molecules-27-03852-s001.zip › molecules-1756430-supplementary.pdf]

molecules

Supplementary Materials

The Allelopathic Activity of Extracts and Isolated from *Spirulina platensis*

Patchanee Charoenying [1,*], Chamroon Laosinwattana [2], and Nawasit Chotsaeng [1,3]

[1] Department of Chemistry, School of Science, King Mongkut's Institute of Technology Ladkrabang, Bangkok 10520, Thailand; nawasit.ch@kmitl.ac.th
[2] Department of Plant Production Technology, School of Agricultural Technology, King Mongkut's Institute of Technology Ladkrabang, Bangkok 10520, Thailand; klchamro@kmitl.ac.th
[3] Integrated Applied Chemistry Research Unit, School of Science, King Mongkut's Institute of Technology Ladkrabang, Bangkok 10520, Thailand
* Correspondence: patchanee.ch@kmitl.ac.th; Tel.: +66-2329-8400 (ext. 6241)

Table S1. Allelopathic effects of E1–E23 fractions from crude ethyl acetate extract on seed germination of Chinese amaranth.

Fractions	% Inhibition on seed germination			
	250 ppm	500 ppm	1000 ppm	2000 ppm
Control	0a	0a	0de	0j
E1	−3.03a	6.06a	3.03c-e	36.36e
E2	3.03a	−3.03a	15.15bc	69.70c
E3	0a	0a	9.09b-d	21.21f-h
E4	0a	3.03a	6.06b-e	30.30ef
E5	−3.03a	3.03a	15.15bc	72.73c
E6	−3.03a	0a	36.36a	84.85b
E7	3.03a	3.03a	15.15bc	66.67c
E8	3.03a	0a	3.03c-e	0j
E9	−3.03a	0a	0de	0j
E10	0a	0a	0de	15.15h
E11	0a	−3.03a	18.18b	72.73c
E12	0a	0a	3.03c-e	90.91ab
E13	0a	3.03a	42.42a	100a
E14	−3.03a	−3.03a	0de	51.52d
E15	0a	3.03	6.06b-e	12.12hi
E16	−3.03a	−3.03	0de	3.03ij
E17	−3.03a	0a	0de	0j
E18	−6.06a	0a	3.03c-e	3.03ij
E19	−6.06a	0a	0de	18.18gh
E20	−3.03a	−6.06a	−6.06e	−6.06j
E21	0a	0a	3.03c-e	15.15h
E22	−6.06a	−6.06a	3.03c-e	27.27e-g
E23	−3.03a	0a	15.15bc	21.21f-h

Values with the same letters in each column there are not significantly different at $p \leq 0.05$

Table S2. Allelopathic effects of E1-E23 fractions from crude ethyl acetate extract on seedling growth of Chinese amaranth.

Fractions	% Inhibition on shoot growth				% Inhibition on root growth			
	250 ppm	500 ppm	1000 ppm	2000 ppm	250 ppm	500 ppm	1000 ppm	2000 ppm
Control	0a	0b-e	0e-g	0h-i	0a-d	0a	0b	0g-i
E1	−2.11ab	−4.38c-g	−14.91i-k	16.21ef	−11.06e-g	−29.05g	−34.67hi	−3.06hi
E2	−13.45cd	−4.38c-g	−11.51h-j	20.10e	−25.53ij	−2.81a-c	0b	43.02d
E3	−16.69d	−14.59i-k	−29.66l	−12.32j	−26.83ij	−27.54fg	−43.22i	−15.88jk
E4	−4.86a-c	−3.73c-f	−18.31jk	7.62f-h	−22.81hi	−28.34g	−23.32fh	7.84fg
E5	−6.00a-c	−14.10h-k	−1.62e-h	87.03b	−1.51a-e	−18.59d-f	−16.68e-g	83.92bc
E6	−0.65ab	10.86a	68.40a	90.28ab	−3.22a-f	0.10a	69.95a	89.95b
E7	−14.42cd	−1.30b-e	10.86b-d	34.04d	−33.97jk	−11.16cd	−3.52b-d	48.74d
E8	−17.67d	−11.18f-i	−11.67h-j	−3.08ij	−18.39g-i	−11.76cd	−15.78d-g	−8.54ij
E9	−14.26cd	−27.55l	−23.01kl	−29.17k	1.61a-c	−18.79d-f	−23.02fh	−26.63l
E10	−21.72d	−15.07i-k	−9.24g-j	0.65g-i	−35.48jk	−21.61fg	−6.63b-e	8.84fg
E11	−34.85e	−21.88kl	−11.83h-j	74.88c	−41.91k	−11.46cd	−8.94b-e	78.89cc
E12	−15.07cd	−12.80g-j	−6.00f-i	85.41b	−13.57f-h	−5.93a-c	−14.67c-g	75.88c
E13	−19.29d	−20.75j-l	−15.88i-k	100a	−9.95d-g	−8.44a-c	−23.52fh	100a
E14	−15.40cd	−9.08e-i	1.13d-g	91.09ab	−18.59g-i	−22.11fg	1.31b	79.90c
E15	−11.18b-d	−11.02f-i	−12.97i-k	11.02eg	−19.50g-i	−10.85cd	−24.02fh	−6.93i
E16	−5.51a-c	−13.13g-j	−13.00i-k	−12.16j	−7.34c-f	−12.06c-e	−26.23gh	−28.04l
E17	−5.51a-c	−5.51d-h	−12.97i-k	−11.99j	−6.33c-f	−21.06e-g	−37.69i	−20.10kl
E18	0.81a	−1.62c-e	−5.19e-i	−3.24ij	−2.71a-f	−5.53a-c	−18.69e-g	4.22gh
E19	−5.83a-c	−3.73c-f	−0.97e-h	39.22d	−5.53b-f	−10.05b-d	−12.66c-f	48.44d
E20	0.49a	−2.43c-f	4.38c-f	8.75f-h	4.92ab	−11.16cd	3.42b	2.21gh
E21	3.57a	3.08a-d	5.35c-e	8.27f-h	5.73a	−2.31a-c	−7.54b-e	14.57f
E22	2.76a	7.13ab	18.48b	35.17d	2.91a-c	−1.01ab	−2.41bc	42.71d
E23	2.43a	3.73a-c	11.67bc	36.30d	−1.51a-e	−10.15b-d	−7.04b-e	32.66e

Values with the same letters in each column there are not significantly different at $p \leq 0.05$

Table S3. Allelopathic effects of E1-E3 fractions from crude ethyl acetate extract on seed germination of barnyardgrass.

Fractions	% Inhibition on seed germination			
	250 ppm	500 ppm	1000 ppm	2000 ppm
Control	0a	0b	0b	0c
E1	−5.26a	0b	5.26b	5.26c
E2	0a	2.63ab	2.63b	0c
E3	5.26a	5.26ab	5.26b	2.63c
E4	0a	2.63ab	0b	0c
E5	−2.63a	2.63ab	7.89b	18.42b
E6	0a	18.42a	71.05a	92.11a
E7	0a	2.63ab	2.63b	0c
E8	2.63a	−2.63b	2.63b	0c
E9	0a	0b	2.63b	5.26c
E10	2.63a	−2.63b	2.63b	7.89bc
E11	0a	0b	5.26b	7.89bc
E12	2.63a	5.26ab	0b	−2.63c
E13	0a	5.26ab	0b	2.63c
E14	5.26a	−2.63b	5.26b	7.89bc
E15	2.63a	5.26ab	5.26b	0c
E16	−5.26a	0b	0b	0c
E17	2.63a	0b	2.63b	−2.63c
E18	5.26a	−2.63b	−2.63b	2.63c
E19	−2.63a	5.26ab	−2.63b	5.26c
E20	0a	5.26ab	2.63b	2.63c
E21	2.63a	2.63ab	0b	0c
E22	5.26a	2.63ab	5.26b	2.63c
E23	0a	5.26ab	5.26b	5.26c

Values with the same letters in each column there are not significantly different at $p \leq 0.05$

Table S4. Allelopathic effects of E1-E23 fractions from crude ethyl acetate extract on seedling growth of barnyardgrass.

Fractions	% Inhibition on shoot growth				% Inhibition on root growth			
	250 ppm	500 ppm	1000 ppm	2000 ppm	250 ppm	500 ppm	1000 ppm	2000 ppm
Control	0ab	0bc	0c	0d	0a	0ab	0c-f	0g
E1	−7.85b	4.78bc	−0.18c	6.84cd	−5.18ab	2.59ab	−5.53fg	0.82g
E2	−3.12ab	−2.43bc	−3.12c	−3.45d	−0.59a	−3.41bc	−10.12g	−17.76h
E3	3.45ab	0.69bc	5.74bc	2.62cd	0.59a	−11.53c	6.12c-e	7.06e-g
E4	3.63ab	6.20bc	6.16bc	6.56cd	7.41a	4.35ab	2.94c-f	6.82e-g
E5	4.73a	6.34bc	12.10b	30.11b	5.18a	1.18ab	25.88b	46.71b
E6	5.24a	16.77a	44.88a	49.47a	−16.47b	−4.71bc	52.94a	72.12a
E7	4.23a	0.64bc	0.41bc	6.29cd	7.06a	−4.24bc	1.18c-f	20d
E8	3.26ab	−1.06bc	−2.02c	5.28cd	3.29a	7.29a	0c-f	15.41d-f
E9	−4.09ab	−3.90c	2.94bc	4.36cd	−3.88ab	2.35ab	0.82c-f	7.18e-g
E10	−3.63ab	3.49bc	5.47bc	1.52cd	−0.24a	2.71ab	6.12c-e	0.71g
E11	3.72ab	5.10bc	3.49bc	4.78cd	3.88a	−3.53bc	6.24c-e	2.47g
E12	2.16ab	3.90bc	7.49bc	5.37cd	5.53a	8.35a	6.24c-e	2.35g
E13	3.17ab	−2.53bc	5.93bc	3.12cd	3.06a	−3.53bc	5.53c-e	−1.18g
E14	5.14a	7.44b	8.22bc	1.33d	8.24a	5.06ab	5.88c-e	−3.65g
E15	0.87ab	1.65bc	4.23bc	6.02cd	3.29a	2.24ab	7.06cd	6fg
E16	3.22ab	5.51bc	5.05bc	6.20cd	5.29a	3.76ab	−1.53d-f	7.53e-g
E17	4.04a	3.40bc	5.93bc	2.66cd	1.06a	2.35ab	5.88c-e	5.76fg
E18	−1.93ab	4.13bc	3.03bc	3.26cd	−1.65a	5.18ab	6c-e	2.71g
E19	2.94ab	5.10bc	2.89bc	8.22cd	8.12a	3.65ab	6.71cd	16.12d-f
E20	4.00a	4.00bc	4.32bc	4.09cd	6a	7.06a	8.24c	17.53de
E21	3.77ab	−1.42bc	2.89bc	10.51cd	7.18a	−0.82ab	26b	31.18c
E22	2.25ab	3.17bc	4.59bc	16.35c	1.88a	−0.12ab	5.18c-e	36.24c
E23	2.89ab	3.26bc	−0.28c	10.28cd	−0.71a	7.88a	−2.82e-g	16.47d-f

Values with the same letters in each column there are not significantly different at $p \leq 0.05$

References for Supporting Materials

33. Charoenying, P.; Chotsaeng, P.; Laosinwattana, C. Effects of *Spirulina platensis* and C-phycocyanin on seed germination and seedling growth of two monocot and dicot plants. *Allelopathy J.*, **2010**, 25, 453–464.
